# P066 When the temporal arteries go quiet: PET-CT reveals relapsing large-vessel giant cell arteritis despite negative ultrasound and biopsy

**DOI:** 10.1093/rap/rkag113.096

**Published:** 2026-11-03

**Authors:** Thet Htar San, Amanee Rajapaksha, Diana Maria Artene, Jon King, Ognjenka Savanovic-Abel

**Affiliations:** University Hospitals Plymouth, Plymouth, United Kingdom; University Hospitals Plymouth, Plymouth, United Kingdom; University Hospitals Plymouth, Plymouth, United Kingdom; University Hospitals Plymouth, Plymouth, United Kingdom; University Hospitals Plymouth, Plymouth, United Kingdom

## Abstract

**Introduction:**

Giant cell arteritis (GCA) is the most common systemic vasculitis in older adults and can present with cranial symptoms, large-vessel involvement, or both. Diagnosis is increasingly supported by vascular ultrasound and advanced imaging modalities. However, relapsing disease may present a diagnostic challenge, particularly when conventional investigations such as temporal artery ultrasound and biopsy are negative. We report a patient with rheumatoid arthritis and previously ultrasound-confirmed cranial GCA who subsequently developed recurrent symptoms suggestive of relapse. Despite negative temporal artery imaging and biopsy findings, positron emission tomography-computed tomography (PET-CT) demonstrated active large-vessel vasculitis, significantly influencing management.

**Case description:**

A woman with seropositive rheumatoid arthritis diagnosed in 2012 was treated with methotrexate, hydroxychloroquine, adalimumab and subsequently rituximab from 2023.

In August 2023, she presented with severe left-sided ear pain, temporal headache, scalp tenderness, jaw claudication and raised inflammatory markers (CRP 96 mg/L, ESR 90 mm/hr). Temporal artery ultrasound demonstrated bilateral halo signs, confirming giant cell arteritis (GCA). She was commenced on prednisolone 60 mg daily with good clinical response.

In March 2024, following a rapid corticosteroid taper, she developed recurrent headache, jaw claudication, temporal tenderness, limb claudication and transient diplopia. Symptoms improved rapidly following reintroduction of high-dose prednisolone, consistent with GCA relapse.

In October 2024, she again developed temporal, jaw and ear pain with scalp tenderness. However, inflammatory markers remained normal, temporal artery ultrasound showed no evidence of arteritis and temporal artery biopsy demonstrated no active inflammation. Symptoms settled without confirmation of active vasculitis.

In February 2026, after completing corticosteroid therapy, she developed severe left temporal headache, scalp tenderness, jaw claudication and ear symptoms similar to her initial presentation. Temporal artery ultrasound demonstrated no temporal artery halo sign, although mild left axillary artery wall thickening with a halo sign was identified. Inflammatory markers were only mildly elevated (CRP 14 mg/L). Concomitant ear swelling and discharge raised concern for infection, and corticosteroids were initially withheld.

Given persistent diagnostic uncertainty, PET-CT was performed. This demonstrated active large-vessel vasculitis, confirming recurrent GCA despite negative temporal artery imaging. Tocilizumab therapy was commenced for treatment of both large-vessel GCA and rheumatoid arthritis.

**Discussion:**

This case highlights the evolving spectrum of giant cell arteritis and the limitations of relying solely on cranial investigations in patients with suspected relapse. The patient initially had classic cranial GCA with a positive temporal artery ultrasound demonstrating bilateral halo signs and an excellent response to corticosteroids.

Subsequent episodes posed a significant diagnostic challenge. During later presentations, symptoms remained highly suggestive of recurrent GCA, including temporal headache, scalp tenderness and jaw claudication. However, temporal artery ultrasound and temporal artery biopsy were negative. Such findings may falsely reassure clinicians, particularly in patients with a previous confirmed diagnosis.

Large-vessel involvement occurs in a substantial proportion of patients with GCA and may persist despite resolution of cranial artery inflammation. Disease activity may shift from cranial vessels to larger arteries including the axillary, subclavian and aortic branches. In this case, mild axillary artery abnormalities on ultrasound prompted further investigation despite inconclusive inflammatory markers and negative temporal artery imaging.

PET-CT proved crucial in establishing the diagnosis by demonstrating active large-vessel vasculitis. The findings altered management and supported initiation of tocilizumab, which offers both disease control and steroid-sparing benefits. The case also illustrates the importance of excluding infection before commencing immunosuppressive therapy, particularly in patients receiving biologic agents and presenting with ENT symptoms.

This report emphasises the need for a high index of suspicion in patients with relapsing symptoms and demonstrates the complementary role of PET-CT in evaluating suspected large-vessel GCA when conventional investigations are inconclusive.

**Key learning points:**

• Giant cell arteritis may evolve from predominantly cranial disease to large-vessel vasculitis over time. Negative temporal artery ultrasound or biopsy does not exclude active GCA, particularly in relapsing disease. Persistent symptoms such as headache, scalp tenderness and jaw claudication should prompt further evaluation even when inflammatory markers are normal or only mildly elevated. Axillary artery abnormalities on ultrasound may provide an important clue to underlying large-vessel involvement. PET-CT is a valuable diagnostic tool when conventional cranial investigations are inconclusive. Infection should be carefully excluded before escalating immunosuppression in patients with suspected vasculitis relapse. Tocilizumab is an effective steroid-sparing therapy for relapsing and large-vessel GCA and may be particularly useful in patients with co-existing rheumatoid arthritis. Multimodal assessment combining clinical evaluation, ultrasound, laboratory markers and advanced imaging is often required to accurately diagnose relapsing GCA.

